# Predictors of outcome in idiopathic rapidly progressive glomerulonephritis (IRPGN)

**DOI:** 10.1186/1471-2369-7-16

**Published:** 2006-11-01

**Authors:** Efstathios Alexopoulos, Lazaros Gionanlis, Ekaterini Papayianni, Elizabeth Kokolina, Maria Leontsini, Dimitrios Memmos

**Affiliations:** 1 Department of Nephrology, 'Hippokration' General Hospital, Papanastasiou 50 Str, Thessaloniki GR-54642, Greece; 2 Department of Nephrology, 'Papanikolaou' General Hospital, Exohi, Thessaloniki GR-57010, tel: 00306945573075, Greece; 3 Department of Pathology, 'Hippokration' General Hospital, Papanastasiou 50 Str, Thessaloniki GR-54642, Greece

## Abstract

**Background:**

Small vessel vasculitides are known to follow a devastating course towards end-stage renal disease, unless treated with immunosuppressive regiments. We investigated the value of clinical, histological and immunohistochemical parameters as predictors of outcome at diagnosis in patients with pauci immune necrotizing glomerulonephritis.

**Methods:**

In 34 patients the percentage and evolution stage of crescents, the presence of glomerular necrosis, the degree or severity of arteriosclerosis, as well as the extent of tubulointerstitial infiltration, interstial fibrosis and tubular atrophy were assessed. Monoclonal antibodies were used to identify infiltrating macrophages, α-SMA(+) and PCNA(+) cells, the expression of integrins α3β1 and LFA-1β, the adhesion molecule ICAM-1, the growth factor TGF-β1 and the terminal complement component C5b-9.

**Results:**

24 pts (70.6%) showed a complete or partial response to the treatment. The follow-up period was 20 ± 22 months. At multivariate analysis, serum CRP (p = 0.024), the intensity of tubular expression of C5b-9 (p < 0.0001) as well as the extent of glomerular and tubular expression of α3β1 integrin (p = 0.001 and 0.008 respectively) independently predicted the response to treatment. The response rate was better in ANCA(+) pts (p = 0.008). The extent of interstitial infiltrate (p < 0.0001), the severity of tubulointerstitial fibrosis (p < 0.0001) and the severity of tubular TGF-β1 expression (p < 0.0001) were independent predictors of long term outcome of renal function.

**Conclusion:**

Patients with ANCA-associated renal vasculitis seem to respond better to the treatment. Acute phase reactants, such as CRP, implying a more intense parenchymal inflammatory reaction, as well as the intensity of the *de novo *expression of C5b-9 and the glomerular and tubular expression of α3β1 integrin predict the response to therapy. The severity of TIN lesions and of the tubulo-interstitial TGF-β1 and C5b-9 expression predict an unfavourable outcome.

## Background

Idiopathic pauci immune rapidly progressive glomerulonephritis (IRPGN) with few or no deposits in immunofluorescence is a form of renal vasculitis that occurs either as a manifestation of a systemic disease, such as Wegener's granulomatosis and microscopic polyangiitis, or as renal limited vasculitis[1]. Since the Chapel Hill Consensus Conference and the association of these diseases with antineutrophil cytoplasmic autoantibodies (ANCA), the reported incidence has raised (~20 cases per million) with geographical and seasonal variations and a known preponderance for older patients[1,2].

The natural course of the disease renders it a "medical emergency", as the decline in renal function is relentless and leads to end stage renal failure in a few weeks or months. Prognosis of IRPGN is a major concern for patients and physicians, especially because treatment with corticosteroids and cyclophosphamide, though effective in controlling disease, is associated with significant morbidity and mortality. Initiating aggressive immunosuppressive therapy in order to regain or conserve independent renal function in patients with IRPGN therefore depends upon the perceived prognosis of the patient.

The identification of specific prognostic factors in IRPGN has been the subject of many studies, which often showed contradictory results [3-8]. Comparison between these studies is difficult due to large differences in study design, inclusion criteria, biopsy-scoring methods, treatment strategies and end-point definitions.

The aim of the present study was to analyze our experience with patients with IRPGN that were diagnosed and followed in one single center. Furthermore, an attempt was made to identify clinical, histological and immunohistochemical findings predictive of response to treatment and outcome.

## Methods

For the purpose of the study clinical and histological data from 34 adult patients (>15 years of age) with biopsy proven focal necrotizing glomerulonephritis and/or glomerular crescents were studied. On immunofluorescence there were few or no immune-deposits. All patients included in the study had sufficient frozen renal tissue for the immunohistological study. Secondary forms of IRPGN were excluded on the basis of history, physical and laboratory examination findings and follow-up. In particular, patients with immune-complex diseases, such as lupus, Henoch Schoenlein purpura, cryoglobulinemia, postinfectious glomerulonephritis and patients with anti-GBM disease were excluded. In addition, exclusion criteria included pregnancy, previous malignancy, known HIV positivity, regular use of drugs that have been implicated in causing RPGN and hepatitis B antigenemia (HBe antigen positivity).

The presence of extrarenal manifestations and the involvement of other organs were systematically recorded. The patients were screened for the presence of ANCA. For the detection of ANCA an indirect immunofluorescence based on Wiik's method was used[9], while major ANCA subtypes (MPO, myeloperoxidase and PR3, proteinase 3) were determined by an Elisa method (Varelisa for MPO- and PR3-ANCA, Pharmacia-Upjohn).

The treatment protocol consisted of methylprednisolone iv boluses at a dose 7–15 mg/kg BW (max. 1 g) daily for three consecutive days followed by oral prednisone at a daily dose of 1 mg/kg BW with progressive tappering. In addition to corticosteroids, oral cyclophosphamide at a daily dose of 1.5–2 mg/kg BW was given in order to maintain WBC count above 3,000/mm^3^. Plasma exchange was added in case of severe renal impairment necessitating dialysis, in patients with pulmonary hemorrhage, or in those with disease progression or persistence of active disease.

Data collection was recorded at follow-up visits at 1, 3, 6 months after discharge and at 6-month interval until the end of the study. In compliance with the Helsinki Declaration, informed verbal consent was obtained from all patients prior to enrollment, allowing for the use of the patient data and of a tissue sample from their biopsy specimens. The study was approved by the Scientific Committee of the 'Hippokration' General Hospital. The authors declare that they have no competing interests.

### Definitions

Normal renal function was defined as plasma creatinine (pCr) ≤ 1.4 mg/dl. Renal insufficiency was defined as persistent rise of pCr >1.4 mg/dl and ESRD was the point at which dialysis treatment was deemed permanent or when pCr remained above or equal to 10 mg/dl. A response to treatment was defined as improvement of renal function observed within 2 months following initiation of treatment. The response was defined as complete when renal function returned to normal, as partial when renal function stabilized at levels not requiring dialysis, while non response was defined when renal function did not improved or deteriorated necessitating replacement treatment. Clinical end-points of the present study were the institution of chronic dialysis, or death of the patient.

### Renal biopsy evaluation

All biopsies were taken at presentation (prior to immunosuppressive treatment initiation) using a standard Trucut needle. In each case, glass slides stained with haematoxylin-eosin, Masson trichrome, periodic acid-Schiff and methenamine silver periodic acid-Schiff (Jones) were available for study. In addition to establishing the diagnosis of IRPGN, the following features were recorded: the percentage of glomeruli with cellular, fibrocellular and fibrous crescents, the percentage of obsolescent and normal glomeruli, the presence of segmental glomerular necrosis. Furthermore, the severity of tubulointerstitial (TIN) fibrosis, the extent of interstitial inflammatory infiltrate, the severity of tubular atrophy and the presence of arteriosclerosis were evaluated and graded on a scale from 0 to 3, according to the pathologist's scoring considering the affected area of the renal parenchyma (grading 0 when absent, 1 when <30% was affected, 2 when 31–60% was affected and 3 when >61% was affected).

### Immunohistochemical study

Snap-frozen tissue was available for immunohistochemistry. The monoclonal antibodies employed were specific for monocyte/macrophages (M718, DAKO), HLA-DR antigen (M704, DAKO), PCNA (M879, DAKO), α-smooth-muscle-actin (αSMA) (M581-DAKO), LFA-1β (783, DAKO), transforming growth factor β1 (TGF-β1) (anti-hLAP, MAB 246, R&D Systems), ICAM-1 (NCL-CD54, Novocastra), and C5b-9 (M777, DAKO). For the purposes of the study an indirect immunoperoxidase technique was used. The glomerular expression of TGF-β1 and C5b-9, the tubulointerstitial expression of TGF-β1 and the expression of C5b-9 and ICAM-1 by the extraglomerular vessels were evaluated and graded semi-quantitatively on a scale from 0 to 3, with the value '0' indicating no expression, '1'mild, '2' medium and '3' intense expression. Tubulointerstitial expression of C5b-9 and ICAM-1 was expressed as the percentage of tubules expressing the antibodies. The glomerular expression of ICAM-1 was evaluated as normal, decreased or increased. The number of glomerular or interstitial monocyte/macrophages, αSMA, LFA-1β and PCNA-positive cells were expressed as the number of cells per glomerular section or per interstitial mm^2^, respectively. HLA-DR expressing cells were not enumerated in the glomeruli because epithelial and endothelial glomerular cells normally are stained by this antibody and, therefore, infiltrating cells cannot be distinguished readily. The number of interstitial HLA-DR (+) cells was expressed as cells per mm^2^. Renal tissue from nephrectomies served as control.

### Statistical analysis

Comparisons of clinical, histological or immunohistochemical data between different subgroups were performed using Student's *t*-test for unpaired data, as well as the one-way ANOVA. Relationships between parametrical parameters were determined using Pearson's correlation coefficient, while Spearman's correlation coefficient was used to identify relationships between non-parametric parameters. Multivariate regression analysis was used to identify independent predicting factors.

Statistical analysis was performed using a computer program package (SPSS 10.1 for Windows, Standard version, ©SPSS.Inc, USA), and a *P *value of <0.05 was considered significant.

## Results

### Patient characteristics

The clinical data of patients are shown in Table 1. Mean pCr at presentation was 6 ± 4.2 mg/dl and mean C-reactive protein (CRP) levels were 72.8 ± 78 mg/l. Eleven patients (32.3%) presented with oligoanuria and 14 (41.2%) required renal replacement treatment (RRT). Plasma exchange treatment was required in 14 of 34 patients (41.2%), the same individuals as required RRT. The mean number of PE sessions was 8.5/patient (range 4–18) and the mean plasma volume exchanged per session was 2.1 lt (range 1–3.5 lt). Twenty-six of our patients (73%) were ANCA (+) and the ratio MPO: PR3 was 1.9:1. Extra-renal manifestations were recorded in 68% of the patients.

**Table 1:** Clinical characteristics of the patients at diagnosis

n	34
Gender (M:F)	17:17 (ratio 1:1)
Age (years)	55.7 ± 17 (range 25–80 years)
ANCA (yes: no)	26:8 (ratio 3,25:1)
Type of ANCA (MPO: PR3)	17:9 (ratio 1,9:1)
Extrarenal manifestations	23 (67.6%)
Respiratory system	19 (55.9%)
Joints	12 (35.3%)
Gastrointestinal system	7 (20.6%)
Skin	5 (14.7%)
Muscles	2 (5.9%)
Genitourinary system	1 (2.9%)
Liver	1 (2.9%)
Plasma creatinine (mg/dl)	6 ± 4.2 (range 0.8–19.4 mg/dl)
Proteinuria (g/24 h)	1.4 ± 0.96 (range 0.2–4.85 g/24 h)
CRP (mg/l)	72.8 ± 78 (range 3.4–272 mg/dl)
Oligoanuria	11 (32.3%)
Dialysis	14 (41.2%)
Plasma exchange	14 (41.2%)
Follow-up (months)	20 ± 22 (range 1–72 months)

Abbrev: MPO, myeloperoxidase; PR3, Proteinase 3; CRP, C-reactive protein

### Histological findings

The mean number of glomeruli per biopsy was 16 ± 9. The majority of the crescents were of the cellular type and in 20 of 34 biopsies (59%) focal glomerular necrosis was observed. Interestingly, no evidence of interstitial fibrosis or tubular atrophy was noted in 20% and 19% of the biopsies respectively. Arteriosclerosis was observed in all but 16% of our patients. In three biopsies there was focal fibrinoid necrosis with infiltration of lymphocytes and macrophages of a middle-sized artery. The histological characteristics are shown in Table 2.

**Table 2:** Analysis of pathology findings

Glomerular				
Total crescents	61 ± 32%			
Cellular crescents	57 ± 44%			
Fibrocellular crescents	17 ± 35%			
Fibrous crescents	12 ± 23%			
Sclerosed glomeruli	28 ± 22%			
Focal necrosis	20/34 (59%)			

Tubulointerstitial				

	Absent	Mild	Medium	Intense

Cellular infiltrates	20%	37%	17%	26%
TIN fibrosis	20%	20%	37%	23%
Tubular atrophy	19%	55%	10%	16%
Arterial sclerosis	16%	34%	22%	22%

Abbrev: TIN, tubulointerstitial

### Immunohistological findings

Analysis of the immunohistological findings is shown in Table 3. Macrophages were localized mainly around the glomerular capillaries or in the crescents, and to a lesser extent in the glomerular mesangium. In the interstitium, macrophage infiltration was observed around the glomerular capsule, especially around the glomerular hilum, as well as around interstitial arterial vessels. Their distribution in the interstitium was similar to that of HLA-DR (+) cells. In addition, clusters of macrophages and, to a lesser extent, HLA-DR (+) cells were accumulated around the tubules. PCNA (+) cells were identified in the mesangium, but also around the glomeruli and the interstitial vessels. α-SMA expressing cells were identified both in the glomeruli and the tubulointerstitium. Glomerular α-SMA (+) cells were mainly localized in the mesangium and in the crescents. It is of interest that a significant number of cells in the Bowman's capsule expressed the α-SMA antigen, but this expression was less impressive compared to the glomerular expression of the antigen. In the tubulointerstitium, α-SMA (+) cells were distributed primarily around the glomeruli, particularly at sites where breaches at the Bowman's capsule were detected, around tubules and extra-glomerular vessels, as well as in heaps in the interstitial space.

**Table 3:** Phenotypical analysis of renal parenchymal cells

	Glomeruli (/glom section)	TIN (/mm2)
Monocyte/macrophages	15 ± 10	547 ± 198
HLA-DR (+) cells	-	1129 ± 782
PCNA (+) cells	33 ± 16	748 ± 196
α-SMA(+)cells	34 ± 16	692 ± 273
LFA-1β (+) cells	15 ± 6	489 ± 142

The glomerular ICAM-1 expression was considered normal, increased or decreased in 37%, 16% and 47% of the cases, respectively. In addition, 37% (± 12%) of the tubules showed *de novo *expression of ICAM-1. TGF-β1 was expressed mainly in the mesangium, in cellular and fibrocellular crescents, but not in fibrous crescents or obsolete glomeruli. In contrast, C5b-9 expression appeared more prominent in fibrous and fibrocellular crescents than in cellular crescents, while 32% of the tubules expressed C5b-9. Glomerular expression of TGF-β1 was noted in 95% of the cases (mild in 11%, moderate in 37% and intense in 47%). Five percent of the glomeruli did not express TGF-β1 at all. In the TIN, 89% of the biopsies showed TGF-β1 expression (mild in 21%, moderate in 47% and intense in 21%) and 11% showed no expression at all. The interstitial TGF-β1 expression was more prominent at sites of dense cellular infiltration. In addition, TGF-β1 expression was also noted in tubular cells, as well as in extra-glomerular vessels.

### Response to the treatment

A total of 24 patients (70.6%) overall responded to treatment. 5/24 (20.8%) showed a complete response to the treatment and 19/24 (79.2%) a partial one. Mean pCr decreased from 2.1 ± 1.1 to 0.9 ± 0.1 mg/dl (p = 0.006) in the patients who responded completely. In patients with partial response to the treatment, pCr levels decreased from 5.4 ± 2.4 to 3.1 ± 1.2 mg/dl (p = 0.004). In particular, renal function in partial responders improved in 5 of 19 and remained stable in 4 of 19. In the remaining 10 patients renal function gradually deteriorated and 4 patients finally reached ESRD at 23, 36, 51 and 61 months after the diagnosis. The mean period of follow-up was 20 months (range 1–72 months, noting that the value of 1 month of follow up was attributed to those patients presenting with dialysis dependent renal failure and not responding to treatment at all).

### Prognostic significance of presenting features

#### I. Clinical

Clinical characteristics at presentation of responders and non- responders are comparatively listed in Table 4. Responders were older and presented with significantly lower levels of blood urea and creatinine at biopsy. CRP levels were significantly lower in patients who responded to the treatment. In particular, mean CRP levels at diagnosis were 14 ± 12 mg/l in completely responders, 52 ± 51 mg/l in those who responded partially (*P *= 0.009) and 143 ± 92 mg/l in non-responders (*P *< 0.0001). Other clinical features did not differ significantly between responders and non-responders. On multivariate analysis, only CRP levels at presentation were predictive of a response to treatment (p = 0.024).

**Table 4:** Clinical and histologic data at presentation

	**Responders N = 24**	**Non responders N = 10**	**p**
**I. Clinical**			

Age (years)	58 ± 16	44 ± 19	0.036
pCreatinine at biopsy (mg/dl)	4.8 ± 2.6	10.2 ± 5.8	0.001
pUrea at biopsy (mg/dl)	105.2 ± 29.8	184 ± 97.5	0.001
CRP (mg/l)	44 ± 49	143 ± 92	<0.0001
ESR (mm/1 h)	112 ± 28	118 ± 47	NS
24 h urinary albumin excretion (g)	1.26 ± 1.1	1.66 ± 0.64	NS

**II. Histologic**			

Crescents (%)	65 ± 32	47 ± 32	NS
Cellular crescents	64 ± 43	49 ± 53	NS
Fibrocellular crescents	8 ± 20	38 ± 52	0.027
Fibrous crescents	12 ± 24	11 ± 2	NS
Obsolete glomeruli	27 ± 20	29 ± 27	NS
Focal necrosis	1.33 ± 0.48	1.60 ± 0.52	NS
TIN infiltration	1.19 ± 1.03	1.22 ± 0.97	NS
Tubular atrophy	0.95 ± 0.72	1.89 ± 1.16	0.011
TIN fibrosis	1.2 ± 0.99	2.22 ± 0.97	0.002
Arterial sclerosis	1.40 ± 1.07	1.67 ± 1.12	0.05

Abbrev: CRP, C-reactive protein, ESR, erythrocyte sedimentation rate, TIN, tubulointerstitial

#### II. Histologic and immunohistologic

**Figure 1:**
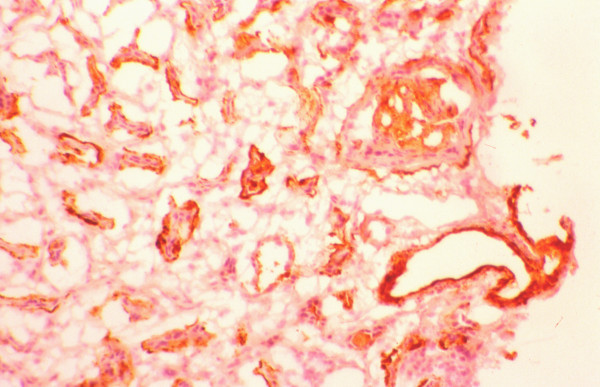
Expression of C5b-9 in a non-responder and a responder.

**Figure 2:**
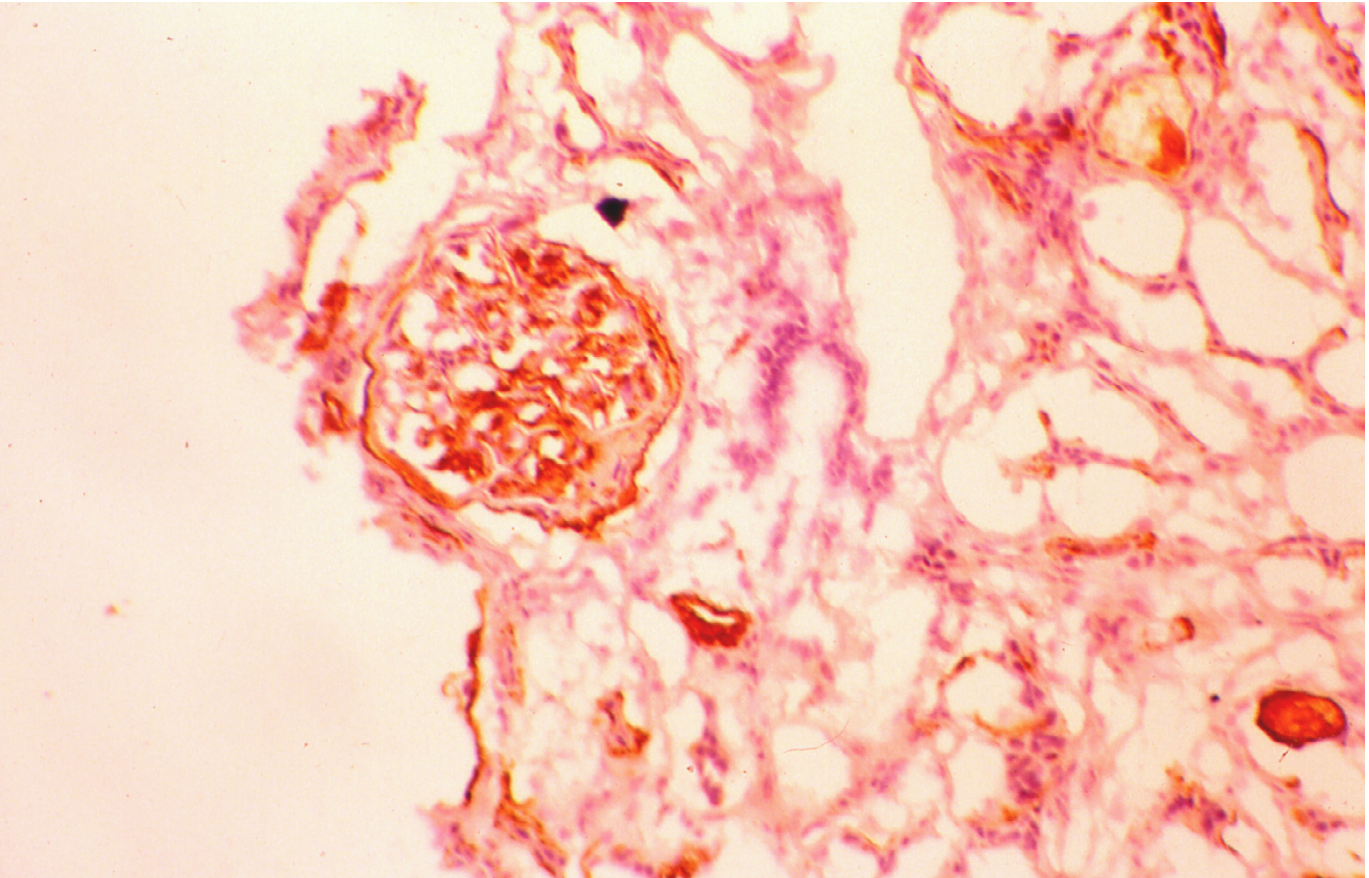
Expression of C5b-9 in a responder.

**Figure 3:**
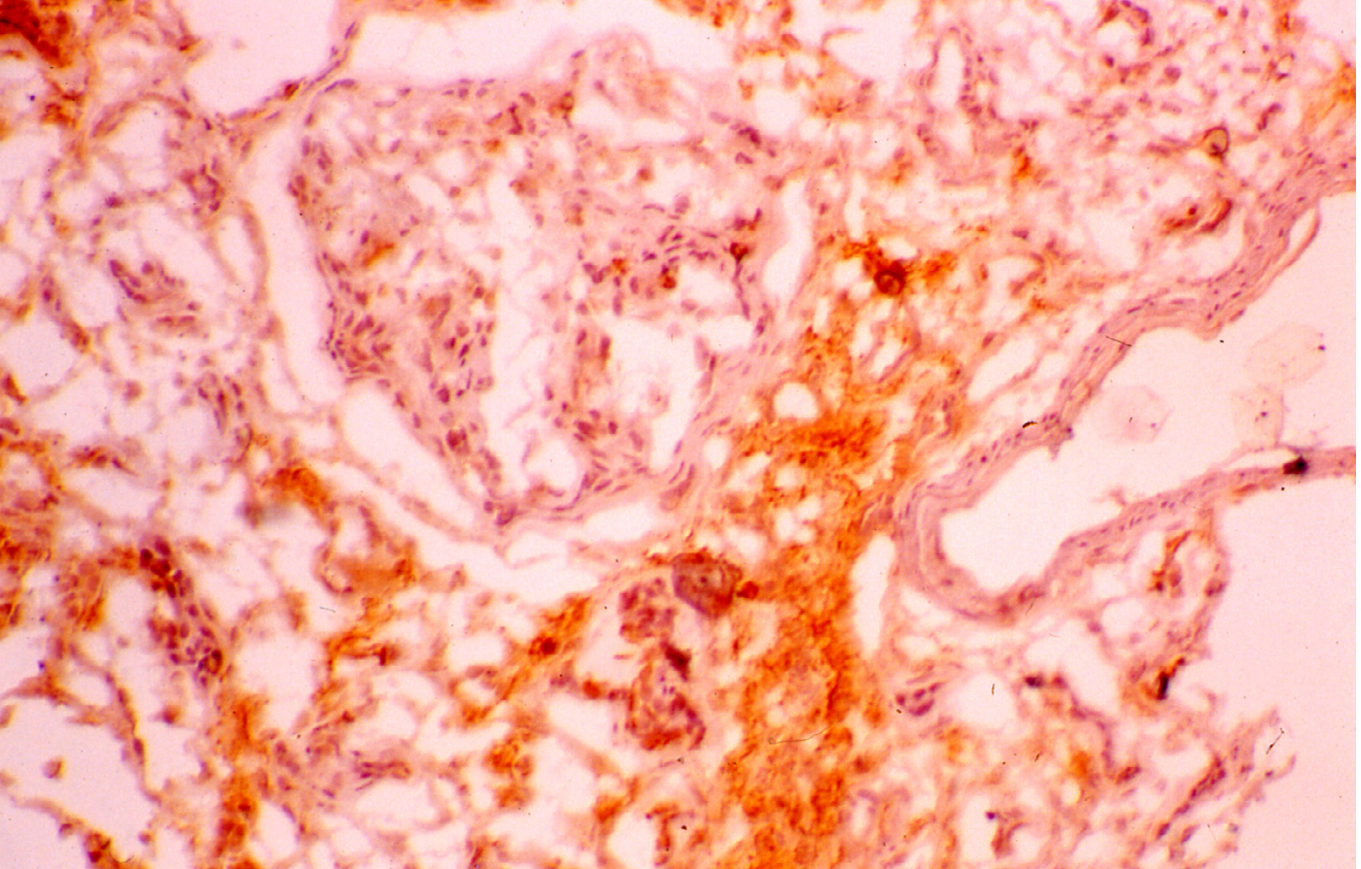
Expression of TGF-β1 in a responder.

**Figure 4:**
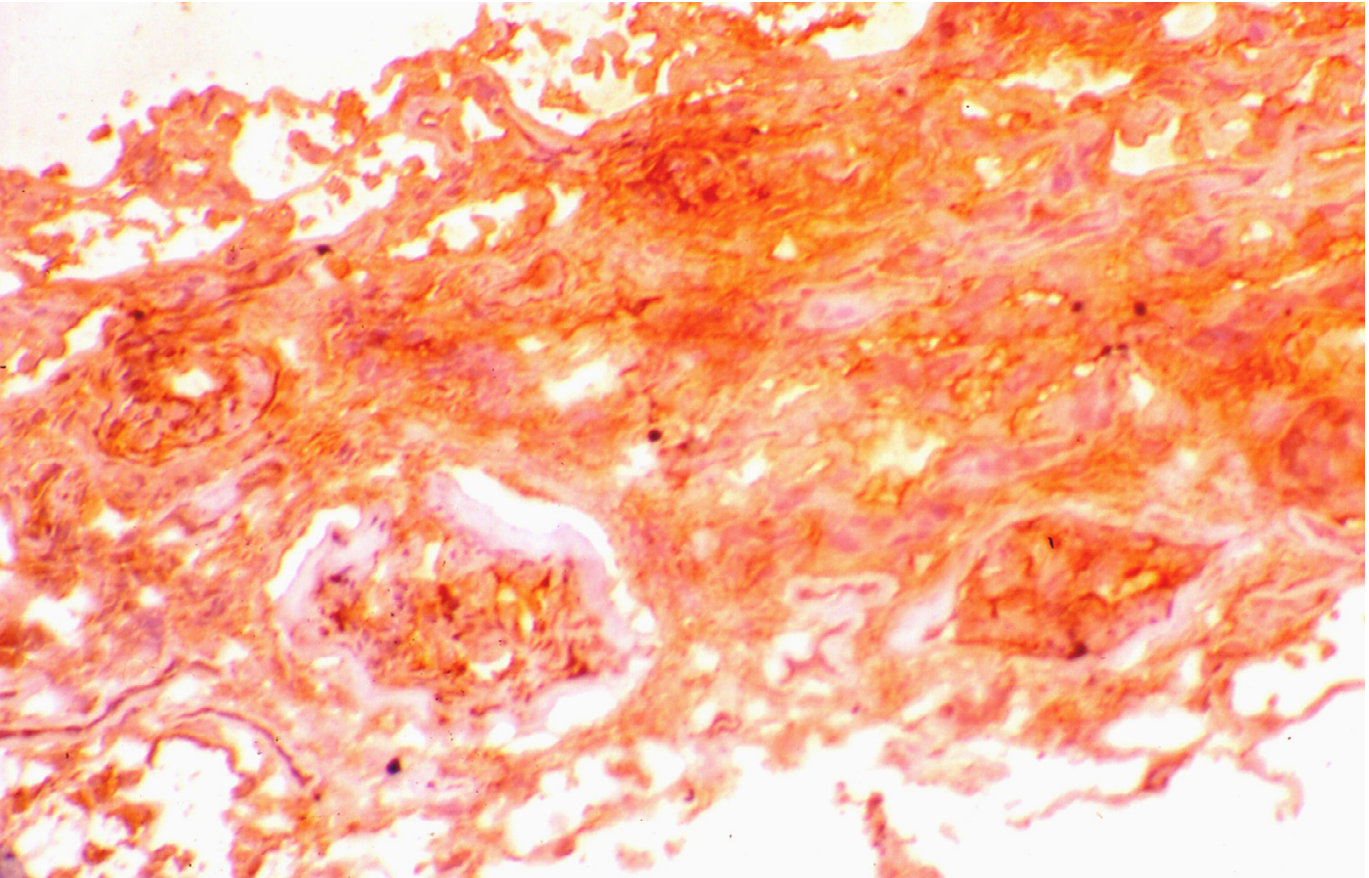
Expression of TGF-β1 in a non-responder.

The mean percentage of glomeruli with crescents was higher in responders (65 ± 32 vs. 47 ± 32), but the difference did not reach the level of statistical significance (Table 4). Also, the distribution of cellular and fibrous crescents was similar between the two groups. However, the percentage of glomeruli with fibrocellular crescents was significantly higher in non-responders (8 ± 20 vs. 38 ± 52, p = 0.027). Normal glomeruli were recorded without statistically significant difference between responders and non-responders. The severity of cellular infiltration of the TIN was similar in both groups. In contrast, non-responders had significantly higher scores of TIN fibrosis, tubular atrophy and arteriosclerosis compared with the group of responders. The numbers of glomerular and interstitial macrophages were similar in both groups (Table 5). Also, the numbers of glomerular and interstitial HLA-DR(+), PCNA(+), α-SMA(+) and LFA-1β(+) cells did not differ significantly between responders and non-responders (table 5). The glomerular expression of ICAM-1 and C5b-9 was similar in both, responders and non-responders, but the percentage of tubules expressing these antigens was significantly higher in non-responders (figures 1, 2). In addition, the glomerular expression of TGF-β1 was significantly higher in non-responders (figures 3, 4), but the intensity of TIN TGF-β1 expression was similar to that of responders. On multivariate analysis, the percentage of tubules expressing C5b-9 (*P *< 0.0001) was shown to be the only independent factor predictive of the response to treatment. The long term outcome of renal function, as defined by the levels of pCr at the end of follow up, correlated with the pCr and CRP levels at presentation (p = 0.006 and p = 0.005, respectively), the severity of TIN infiltration and fibrosis (p < 0.0001), the degree of tubular atrophy and arterial sclerosis (p < 0.0001 and p = 0.049, respectively). The degree of TGF-β1 and C5b-9 expression in the TIN showed significant correlation with the final pCr (p = 0.002 and p < 0.0001, respectively). On multivariate analysis however, only the severity of TIN infiltration and fibrosis and the expression of TGF-β1 in the tubulointerstitium were independent predictors of the long-term outcome.

**Table 5:** Immunohistological findings at presentation

	**Responders N = 24**	**Non responders N = 10**	**p**
**I. Glomeruli**			

PCNA(+) cells	38 ± 16	31 ± 16	NS
α-SMA(+)cells	30 ± 16	38 ± 17	NS
Monocytes/macrophages	16 ± 11	15 ± 8	NS
LFA-1β(+)cells	14 ± 5	22 ± 8	NS
TGF-β1 expression	1.0 ± 0.63	2.2 ± 0.84	0.046
ICAM-1 expression	1.67 ± 0.78	1.71 ± 0.75	NS
C5b-9 expression	2.14 ± 0.95	2.6 ± 0.54	NS

**II. Tubulointerstitium**			

HLA-DR(+) cells	986 ± 702	1484 ± 1022	NS
PCNA(+)cells	845 ± 145	686 ± 160	NS
α-SMA(+)cells	732 ± 275	497 ± 224	NS
Monocytes/macrophages	580 ± 222	473 ± 144	NS
LFA-1β(+)cells	512 ± 151	460 ± 132	NS
ICAM-1 in tubules	29.3 ± 9.5	48.8 ± 4.9	<0.0001
C5b-9 in tubules	29.6 ± 8.5	38.9 ± 9.1	0.05
TGF-β1 in interstitium	1.57 ± 0.94	2.4 ± 0.55	NS
TGF-β1 in tubules	1.78 ± 0.80	2.4 ± 0.89	NS
ICAM-1in vessels	1.27 ± 0.46	1.10 ± 0.01	NS
C5b-9 in vessels	1.06 ± 0.26	1.01 ± 0.09	NS

#### III. The effect of ANCA

ANCA (+) patients tended to respond more successfully to the treatment as compared with ANCA (-) patients (80% vs. 57%, p = 0.008). In addition, there was a tendency towards a better response to the treatment in PR3 (+) patients than MPO (+) patients (89% vs. 75%) (Table 6), although this difference did not reach the level of significance. A comparison between the two groups of patients showed that ANCA (+) patients were older (60 ± 13 vs. 42 ± 20, p = 0.005), and had more severe proteinuria at biopsy (2.21 ± 1.12 vs. 1.17 ± 0.77, p = 0.005) (Tables 7, 8). In addition, ANCA (+) patients had more frequently focal glomerular necrosis in their glomeruli (65.4% vs. 37.5%). Also, ANCA (+) patients had more intense glomerular TGF-β1 expression (1.14 ± 0.69 vs. 2.25 ± 0.96, p = 0.050) and higher numbers of PCNA (+) cells and macrophages in the TIN (809 ± 170 vs. 581 ± 181, p = 0.040 and 605 ± 194 vs. 581 ± 181, p = 0.041, respectively). Interestingly, MPO (+) patients presented with less TIN PCNA (+) cell infiltration (756 ± 166 vs. 947 ± 92, p = 0.049) and TGF-β1 expression than PR3 (+) patients (1.45 ± 0.7 vs. 2.67 ± 0.6, p = 0.017) (Tables 9, 10).

**Table 6:** Response to treatment according to ANCA status

	ANCA(+) n = 26	ANCA(-) n = 8	MPO(+)-ANCA n = 17	PR3(+)-ANCA n = 9
Response	20/26 (77%)	4/8 (50%)	12/17 (71%)	8/9 (89%)
complete	16/26 (62%)	3/8 (38%)	9/17 (53%)	1/9 (11%)
partial	4/26 (15%)	1/8 (12%)	3/17 (17%)	7/9 (78%)
No response	6/26 (23%)	4/8 (50%)	5/17 (29%)	1/9 (11%)

See text for details

**Table 7:** Clinical and histologic differences between ANCA (+) and ANCA (-) patients

	**ANCA (+) N = 26**	**ANCA (-) N = 8**	**p**
**I. Clinical**			

Age (years)	60 ± 13	42 ± 20	0,005
pCreatinine at biopsy (mg/dl)	6.11 ± 4.7	5.7 ± 1.8	NS
pUrea at biopsy (mg/dl)	128.3 ± 69.3	109.4 ± 25.5	NS
CRP (mg/l)	64.7 ± 75	99 ± 86	NS
ESR (mm/1 h)	116 ± 28	108 ± 44	NS
24 h urinary albumin excretion (g)	2.21 ± 1.12	1.17 ± 0.77	0,005

**II. Histologic**			

Crescents (%)	63 ± 33	52 ± 32	NS
Cellular crescents	59 ± 44	51 ± 47	NS
Fibrocellular crescents	11 ± 26	51 ± 47	NS(0.06)
Fibrous crescents	12 ± 24	11 ± 21	NS
Obsolete glomeruli	25 ± 23	35 ± 19	NS
Focal necrosis	1.34 ± 0.48	1.62 ± 0.52	NS
TIN infiltration	1.36 ± 1.13	1.87 ± 0.99	NS
Tubular atrophy	1.13 ± 0.97	1.5 ± 0.93	NS
TIN fibrosis	1.4 ± 1.1	1.59 ± 0.5	NS
Arterial sclerosis	1.79 ± 1.06	2.25 ± 0.96	0.05

Abbrev: CRP, C-reactive protein, ESR, erythrocyte sedimentation rate, TIN, tubulointerstitial

**Table 8:** Differences in immunohistochemical findings between ANCA (+) and ANCA (+) patients

	**ANCA (+) N = 26**	**ANCA (-) N = 8**	**p**
**I. Glomeruli**			

PCNA(+) cells	36 ± 16	26 ± 16	NS
α-SMA(+)cells	34 ± 15	32 ± 19	NS
Monocytes/macrophages	15 ± 10	14 ± 10	NS
LFA-1β(+)cells	15 ± 6	10 ± 4	NS
TGF-β1 expression	1.14 ± 0.69	2.25 ± 0.96	0.05
ICAM-1 expression	1.53 ± 0.64	2.25 ± 0.96	NS(0.06)
C5b-9 expression	2.27 ± 0.96	2.25 ± 0.5	NS

**II. Tubulointerstitium**			

HLA-DR(+) cells	1207 ± 831	736 ± 300	NS
PCNA(+)cells	809 ± 170	581 ± 181	0.041
α-SMA(+)cells	728 ± 260	556 ± 318	NS
Monocytes/macrophages	570 ± 204	452 ± 156	NS
LFA-1β(+)cells	485 ± 149	517 ± 86	NS
ICAM-1 in tubules	34.2 ± 11.4	45.5 ± 12.5	NS
C5b-9 in tubules	32,3 ± 9,7	32,7 ± 8.16	NS
TGF-β1 in interstitium	1.73 ± 0.8	2 ± 1.41	NS
TGF-β1 in tubules	1.87 ± 0.83	2.25 ± 0.96	NS
ICAM-1in vessels	1,21 ± 0,42	1.0 ± 0.01	NS
C5b-9 in vessels	1.06 ± 0.25	1.01 ± 0.09	NS

**Table 9:** Clinical and histologic differences between MPO-ANCA (+) and PR3-ANCA (+) patients

	**MPO-ANCA (+) N = 17**	**PR3-ANCA (+) N = 9**	**p**
**I. Clinical**			

Age (years)	63 ± 10	55 ± 17	NS
pCreatinine at biopsy (mg/dl)	5.9 ± 4.5	6.5 ± 5.3	NS
pUrea at biopsy (mg/dl)	123 ± 56	138 ± 92	NS
CRP (mg/l)	54 ± 63	84 ± 95	NS
ESR (mm/1 h)	116 ± 34	116 ± 16	NS
24 h urinary albumin excretion (g)	1.24 ± 0.81	1.04 ± 0.73	NS

**II. Histologic**			

Crescents (%)	61 ± 35	67 ± 28	NS
Cellular crescents	56 ± 44	66 ± 46	NS
Fibrocellular crescents	13 ± 30	8 ± 17	NS
Fibrous crescents	10 ± 21	15 ± 31	NS
Obsolete glomeruli	28 ± 22	20 ± 24	NS
Focal necrosis	1.41 ± 0.5	1.22 ± 0.44	NS
TIN infiltration	1.53 ± 1.12	1.0 ± 1.15	NS
Tubular atrophy	1.4 ± 0.98	0.62 ± 0.74	NS
TIN fibrosis	1.5 ± 1.09	1.07 ± 1.09	NS
Arterial sclerosis	1.94 ± 1.0	1.5 ± 1.19	NS

Abbrev: CRP, C-reactive protein, ESR, erythrocyte sedimentation rate, TIN, tubulointerstitial

**Table 10:** Differences in immunohistochemical findings between MPO-ANCA (+) and PR3-ANCA (+) patients

	**MPO-ANCA (+) N = 17**	**PR3-ANCA (+) N = 9**	**p**
**I. Glomeruli**			

PCNA(+) cells	28 ± 14	47 ± 12	NS
α-SMA(+)cells	36 ± 12	32 ± 23	NS
Monocytes/macrophages	17 ± 12	12 ± 3	NS
LFA-1β(+)cells	14 ± 5	21 ± 8	NS
TGF-β1 expression	1.0 ± 0.7	1.5 ± 0.7	NS
ICAM-1 expression	1.5 ± 0.67	1.66 ± 0.58	NS
C5b-9 expression	2.2 ± 1.08	2.5 ± 0.58	NS

**II. Tubulointerstitium**			

HLA-DR(+) cells	1293 ± 894	866 ± 467	NS
PCNA(+)cells	757 ± 166	947 ± 92	0.041
α-SMA(+)cells	702 ± 222	779 ± 348	NS
Monocytes/macrophages	550 ± 181	614 ± 264	NS
LFA-1β(+)cells	469 ± 157	552 ± 106	NS
ICAM-1 in tubules	35.4 ± 10.8	29.4 ± 15	NS
C5b-9 in tubules	30.6 ± 9,4	37.3 ± 10.2	NS
TGF-β1 in interstitium	1.45 ± 0.7	2.67 ± 0.6	0.017
TGF-β1 in tubules	1.67 ± 0.78	2.25 ± 0.96	NS
ICAM-1in vessels	1,18 ± 0,4	1.3 ± 0.57	NS
C5b-9 in vessels	1.08 ± 0.29	1.0 ± 0.07	NS

## Conclusion

The results of the present study showed that 70 per cent of our patients with IRPGN and moderate to severe disease responded to the treatment. Combined treatment with corticosteroids and cyclophosphamide induces remission in 75–80% of patients with renal vasculitis according to various reports [10-12]. The relatively lower rate of response that was achieved in this study may be related to late referral of most patients, since mean plasma creatinine at presentation was high enough (~6 mg/dl) and 41% of the patients were dialysis dependent. This study corresponds with other reports according to which the degree of renal function impairment at presentation inversely correlates with the response to the treatment and the subsequent renal outcome [3-12]. In order to determine the influence of other parameters on the response to the treatment, we tested several clinical and pathological factors independent of the level of initial renal function.

The percentage of crescents and particularly the percentage of cellular crescents did not differ between responders and non-responders. Also the prevalence of fibrinoid necrosis, which plays a role in the evolution of crescents, was similar in both groups. These findings are in agreement with previous studies reporting that both of these histological lesions may be regarded as signs of active disease and hence are more likely to be reversed with the treatment [5-10]. Contrary to other studies, normal glomeruli were observed at even scores between the two groups, denoting that the percentage of normal glomeruli was insignificant as a determinant of renal function impairment and prognosis of treatment response. In support of the histological findings, the numbers as well as the type of cells (macrophages, myofibroblast-like cells, proliferating cells, LFA-1β expressing cells) infiltrating the glomeruli were remarkably similar in both groups.

Interestingly, fibrocellular crescents, in contrast to cellular crescents, were more frequently found in non-responders, while the proportion of fibrous crescents was identical in the two groups. This means that these lesions are somehow related to renal function recovery. Our findings indicate that there may be a certain point of no return after which a crescentic glomerulus loses its potential of recovering to a histologically normal glomerulus. In untreated patients the transformation of a cellular crescent to a fibrocellular crescent seems to be a matter of time, since they were found in patients with more advanced chronic lesions in their biopsies indicating a more protracted course of their disease[13,14]. The exact mechanisms incriminated in the development and evolution of this transformation in humans are not clear. However, it seems that glomerular TGF-β1 may play a crucial role since its expression was significantly more pronounced in non-responders, despite the similar number of fibrous crescents.

The severity of interstitial fibrosis and tubular atrophy as well as the degree of arteriosclerosis were higher in non-responders and this finding confirms previous observations [4-10]. Of interest is that the numbers of interstitial macrophages, the numbers of activated or proliferating interstitial cells as well as the numbers of interstitial myofibroblasts (αSMA (+) cells) did not differ between responders and non-responders. In addition, TGF-β1 expression in the tubulointerstitium was found to be similar in both groups. Our results suggest that other factors, such as the activation of tubular cells indicated by the de novo expression of ICAM-1 and C5b-9, may be of importance, as is the case with other forms of glomerular diseases [15-17]. Activated tubular cells are capable of synthesizing numerous cytokines, adhesion molecules, complement components and extracellular matrix[18,19]. The degree of expression of C5b-9 by the tubules also seems to be highly predictive of subsequent renal outcome in different glomerulopathies [20-23]. Interestingly, in our study the degree of this expression was the only independent factor that predicted the response to the treatment.

ANCA (+) patients were older, had significantly greater proteinuria compared to ANCA (-) patients and they showed more prominent glomerular expression of TGF-β1 as well as more numerous proliferating interstitial cells. Also, fibrinoid necrosis was more commonly found in ANCA (+) than in ANCA (-) patients. An increased likelihood between ANCA positivity and glomerular necrosis has never been reported. Whether ANCA and particularly PR3-ANCA, as our findings could suggest, bind to the endothelium and increase the possibility of glomerular necrosis should be further investigated. Irrespective of the exact mechanisms, our findings indicate that increased proteinuria in ANCA (+) patients may be associated with early deposition of TGF-β1 in the glomeruli and, hence, predispose to an increased likelihood of sclerosis. Whether proteinuria is the cause of the increased interstitial cell proliferation in the ANCA (+) group of patients is not known. If this is the case, however, it does not seem to involve tubular cell activation, as the expression of ICAM-1 and C5b-9 by the tubules was similar between ANCA (+) and ANCA (-) patients. Certainly, the number of patients included in this study is small and our results need to be confirmed by larger trials, where the two groups will be evenly represented. However, these findings may help in the establishment of new treatment strategies for this disease.

Eighty percent of ANCA (+) patients showed a complete (62%) or partial response (15%) to the treatment, while only 50% of the ANCA (-) group showed a similar response (38% complete and 12% partial response). The reason for this difference is not clear and relevant data are scarce in the literature. For example, Adu et al reported patients with microscopic polyangiitis with no clinical or laboratory differences between ANCA (+) and ANCA (-) patients[24]. Others, however, found fewer relapses and a more favorable outcome in ANCA (-) patients[25,26]. Certainly different diagnostic criteria and treatment policies may affect the interpretation of various reports. However, the increased proliferation of interstitial cells found in ANCA (+) patients in our study may reflect an increased susceptibility of these patients to treatment and this could explain in part the favorable response of these patients. In support of this view, PR3-ANCA (+) patients who had more numerous interstitial proliferating cells compared to MPO-ANCA (+) patients showed also a tendency towards a better response to the treatment (89% vs. 75%). Several in vitro studies have shown that peripheral blood mononuclear cells proliferate more actively with PR3 than with MPO [27-29]. In addition IgG fractions from anti-PR3 (+) patients were more potent activators of the respiratory burst and degranulation than IgG fractions from anti-MPO (+) patients[5,27,28]. These observations suggest that cell activation markers may contribute to the differences in the response to the treatment between anti-PR3 and anti-MPO associated renal vasculitis. This may alter the perspective regarding the intensity and the duration of the immunosuppressive therapy in these patients. However, our results need to be confirmed by larger studies before they establish a new parameter to tailor treatment.

In conclusion, baseline renal function is the best clinical predictor of a response to therapy in patients with IRPGN. Fibrocellular, but not cellular, fibrous crescents or fibrinoid necrosis seem to be associated with a poorer response to therapy. Chronic interstitial lesions as well as the extent of tubular cell activation may also carry an increased risk for no response. ANCA (+) patients and particularly PR3-ANCA (+) seem to respond better to the treatment. This may be related to the increased rate of interstitial cell proliferation, but this contention should be further investigated.

## Competing interests

The author(s) declare that they have no competing interests.

## Authors' contributions

EK carried out the ANCA subtyping, ML performed the evaluation of the histologic findings, EP participated in the design of the study, DM helped in the study's design and coordination, EA conceived of and coordinated the study and drafted the manuscript, LG performed and evaluated the immunohistochemistry and the statistical analysis and helped to draft the manuscript

## Pre-publication history

The pre-publication history for this paper can be accessed here:


